# Cytoprotective Effect of Hispidin against Palmitate-Induced Lipotoxicity in C2C12 Myotubes

**DOI:** 10.3390/molecules20045456

**Published:** 2015-03-27

**Authors:** Jun Myoung Park, Jong Seok Lee, Jeong Eun Song, Ye Chan Sim, Suk-Jin Ha, Eock Kee Hong

**Affiliations:** 1 Department of Bioengineering and Technology, Kangwon National University, Chuncheon, Gangwon-do 200-701, Korea; E-Mails: three0313@nate.com (J.M.P.); jongseoklee78@gmail.com (J.S.L.); thdwl92@hanmail.net (J.E.S.); simyechan@naver.com (Y.C.S.); sjha@kangwon.ac.kr (S.-J.H.); 2 National Institute of Biological Resources, Incheon 404-708, Korea

**Keywords:** hispidin, oxidative stress, type 2 diabetes, C2C12 skeletal muscle cells, *Phellinus linteus*

## Abstract

It is well known that *Phellinus linteus*, which produces hispidin and its derivatives, possesses antioxidant activities. In this study, we investigated whether hispidin has protective effects on palmitate-induced oxidative stress in C2C12 skeletal muscle cells. Our results showed that palmitate treatment in C2C12 myotubes increased ROS generation and cell death as compared with the control. However, pretreatment of hispidin for 8 h improved the survival of C2C12 myotubes against palmitate-induced oxidative stress via inhibition of intracellular ROS production. Hispidin also inhibited palmitate-induced apoptotic nuclear condensation in C2C12 myotubes. In addition, we found that hispidin can suppress cleavage of caspase-3, expression of Bax, and NF-κB translocation. Therefore, these results suggest that hispidin is capable of protecting C2C12 myotubes against palmitate-induced oxidative stress.

## 1. Introduction

Insulin resistance is recognized as a major risk factor for type 2 diabetes mellitus, and it is characterized by the reduced ability of insulin to regulate glucose homeostasis in target tissues, such as muscle, liver, and adipose [1,2]. Among these target tissues, skeletal muscle is responsible for more than 75% of glucose disposal in response to insulin in the post-prandial state [3,4]. Therefore, skeletal muscle is commonly viewed as a critical component of whole-body insulin resistance. 

It is well known that elevation in plasma free fatty acids (FFAs) levels is commonly associated with impaired insulin-mediated glucose uptake in skeletal muscles [5,6]. Especially, chronic exposure to FFA leads to lipotoxicity and, in turn, apoptosis in skeletal muscle cells [7]. Recent studies demonstrated that lipotoxicity resulting from FFA-induced oxidative stress contributes to whole-body insulin resistance [8,9]. Oxidative stress impairs various cellular functions and plays important roles in many diseases such as inflammation, aging, and diabetes. Oxidative stress results from excessive production of reactive oxygen species (ROS) and causes cellular injury through nonspecific modification and disruption of proteins, phospholipids, and DNA [10]. Skeletal muscle is particularly susceptible to oxidative stress because it requires a large amount of oxygen for muscle action and is post-mitotic, and thus, capable of accumulating oxidative damage over time [11]. Because skeletal muscle is responsible for more than 75% of glucose disposal in response to insulin in the post-prandial state, it is very important to protect skeletal muscle from FFA-induced lipotoxicity.

Apoptosis is the process of programmed cell death, which may occur in multicellular organisms [12,13]. This process is the controlled destruction of a cell that is either infected, defective in a process, no longer needed, or too old [14]. Apoptosis is accompanied with cell membrane shrinkage, nuclear fragmentation, chromatin condensation, and blebbing. Apoptosis is triggered by multiple signal pathways and regulated by multiple complicated extrinsic and intrinsic ligands [15]. Among them, the most dangerous factor is oxidative stress generated by ROS. Elevated levels of intracellular ROS have been implicated in expression of Bcl-2 family proteins such as Bax, Bcl-2, and Bcl-xL [16,17,18]. Excessive generation of ROS, such as superoxide anion and hydroxyl radical, initiates the mitochondria-mediated apoptotic pathway by altering the expression of Bcl-2 family proteins, thereby increasing the mitochondrial transmembrane potential, affecting mitochondrial membrane integrity, and releasing cytochrome c [18]. Release of cytochrome c from mitochondria can lead to the formation of an apoptosome, a multi-protein complex consisting of cytochrome c, apoptotic peptidase activating factor 1 (Apaf-1), procaspase-9, and ATP [19]. Apoptosome assembly is required for the activation of apical or initiator procaspases, which activate executioner procaspase dimers, such as procaspase (pc)-3 or pc-7. Activated caspases then target cellular proteins for proteolysis in a process that kills the cell [20,21].

*Phellinus linteus*, a well-known fungus of the genus *Phellinus* in the family Hymenochaetaceae, has been widely used as folk medicinal mushroom for long time [22,23]. *Phellinus linteus* is known to be abundant in polysaccharides, and several polyphenol compounds, such as hispidin and hispidin analogues, have been isolated from the cultured mycelia [24,25]. Among them, hispidin has been shown to be a protein kinase C inhibitor and possess antioxidant and anticancer activities [26]. Accordingly, in this study, we investigate whether the antioxidant activity of hispidin has a cytoprotective effect against palmitate-induced lipotoxicity.

## 2. Results and Discussion

### 2.1. Optimal Concentration of Hispidin and Palmitate for Experiments

**Figure 1:**
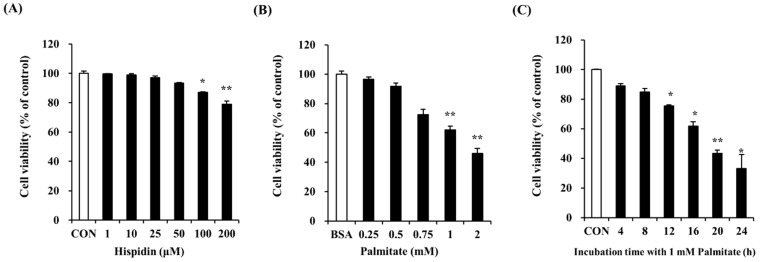
Effect of hispidin and palmitate on C2C12 myotubes viability. (**A**) Cytotoxic effect of hispidin on C2C12 myotubes. Differentiated cells were treated with various concentrations of hispidin for 24 h. The cytotoxicity of palmitate was determined after exposure to various concentrations of palmitate for 16 h; (**B**) and different exposure times with 1 mM palmitate; (**C**). C2C12 myotubes viability was measured by MTT assay. The cells treated with only BSA conditioned medium were used as a control. Palmitate was administered by conjugating it to BSA (fatty acid free). Data represent the mean ± standard error of three independent experiments. * *p* < 0.05, ** *p* < 0.01, significantly different from the control group or only BSA-treated group. CON, control; BSA, bovine serum albumin.

Dose-response experiments were carried out in order to determine the maximum non-toxic concentration of hispidin. MTT assays showed that there was no significant change in cell viability after treatment with hispidin at concentrations from 1 to 100 μM (Figure 1A). At 200 μM hispidin, cell viability was approximately 80%. Therefore, subsequent experiments were conducted at concentrations of up to 200 µM. The cytotoxicity of palmitate was studied in an MTT assay by treating C2C12 myotubes for 16 h. As shown in Figure 1B, concentrations of palmitate ≥ 0.25 mM dramatically reduced cell viability, and cell death was about 61% when treated with 1 mM palmitate. To examine the time-dependent effect of 1 mM palmitate, cell viability was measured after different exposure time to 1 mM palmitate. As shown in Figure 1C, the palmitate concentration (1 mM) and exposure time (16 h) that showed cell viability of about 61% were selected for the next experiments.

### 2.2. Cytoprotective Effect of Hispidin against Palmitate-Induced Cell Death in C2C12 Myotubes

**Figure 2:**
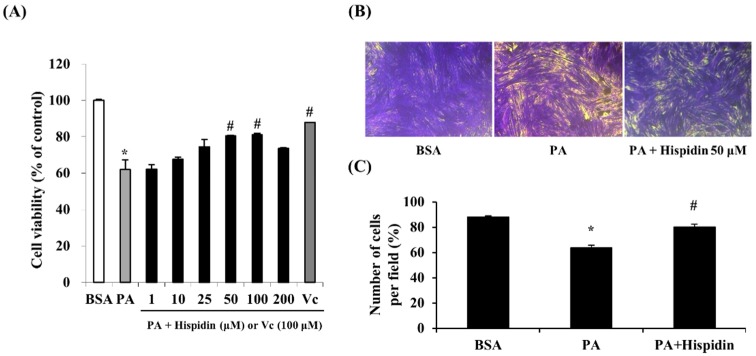
Protective effect of hispidin in palmitate-damaged C2C12 myotubes. (**A**) Effect of hispidin on palmitate-induced cell death in C2C12 myotubes. Cells were treated with 1 to 200 μM hispidin for 8 h, and then 1 mM palmitate was added for 16 h. Cell viability was determined by the MTT assay. Data represent the mean ± standard error of three independent experiments. * *p* < 0.05, as compared with only BSA group, ^#^ *p* < 0.05, as compared with PA group. (**B**) Effect of hispidin on palmitate-induced cell loss in C2C12 myotubes. Cells were treated with hispidin 50 μM for 8 h, and then 1 mM palmitate was added for 16 h. Cell loss was determined by crystal violet staining. (**C**) The number of myotubes per photograph was counted after crystal violet staining using the Image J densitometry software program. The cells treated with only BSA conditioned medium were used as a control. Palmitate was administered by conjugating it to BSA (fatty acid free). BSA, bovine serum albumin; PA, palmitate.

In order to assess the protective effect of hispidin on palmitate-damaged C2C12 myotubes, MTT assays were performed. Compared with the untreated cells, 1 mM palmitate caused a significant reduction in cell viability by 62.1% (Figure 2A). However, pretreatment with hispidin (from 1 to 100 μM) for 8 h in C2C12 myotubes resulted in the prevention of cell death induced by palmitate. Pre-treatment with hispidin at a concentration of 50 μM restored cell viability to 80.4%, relative to the control. To confirm the effect of hispidin on palmitate-induced atrophy and loss in C2C12 myotubes, morphological studies were conducted by crystal violet staining. Myotubes treated with 1 mM palmitate caused a significant decrease in myotubes. However, pre-treatment with hispidin 50 μM attenuated the palmitate-mediated loss of C2C12 myotubes (Figure 2B).

### 2.3. Effect of Hispidin on Palmitate-Induced Oxidative Stress

**Figure 3:**
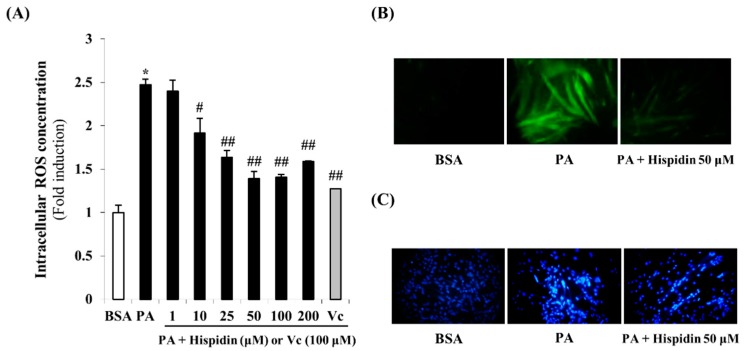
Protective effect of hispidin on palmitate-induced oxidative stress in C2C12 myotubes. (**A**) Intracellular ROS scavenging activity of hispidin on palmitate-treated C2C12 myotubes. Cells were treated with 1 to 200 μM hispidin or 100 μM Vitamin C (Vc) for 8 h, and then 1 mM palmitate was added for 16 h. The intracellular ROS generation was measured by DCF-DA methods. Data represent the mean ± standard error of three independent experiments. * *p* < 0.05, as compared with only BSA group, ^#^ *p* < 0.05, as compared with PA group, ^##^ *p* < 0.05, as compared with PA group. (**B**) Fluorescence microscopic images of cells stained for ROS (magnification 400×). (**C**) DNA condensation of C2C12 myotubes was detected by Hoechst 33342 staining. DNA condensation in cells was stained with Hoechst 33342 solution and visualized using a fluorescent microscope (magnification 100×). BSA, bovine serum albumin; PA, palmitate; Vc, Vitamin C.

To investigate the intracellular ROS scavenging activity of hispidin, the ROS sensitive fluorescent probe, H_2_DCF-DA was used. As shown in Figure 3A, cells treated with palmitate showed a 2.5-times greater induction in intracellular ROS levels. However, pre-treatment with hispidin for 8 h significantly reduced intracellular ROS concentration. In the fluorescent microscope images, the fluorescence intensity was enhanced in palmitate-treated C2C12 myotubes. However, pre-treatment with hispidin for 8 h greatly decreased the fluorescence intensity (Figure 3B). These results suggest that hispidin might prevent palmitate-induced oxidative stress through ROS scavenging activity. In order to confirm the DNA condensation in palmitate-induced oxidative stress in C2C12 myotubes, DNA of C2C12 myotubes was stained Hoechst 33342. Cells treated with 1 mM palmitate for 16 h showed typical characteristics of apoptosis, including the condensation of chromatin, the nuclear shrinkage, and the appearance of a few apoptotic bodies. However, in 50 μM hispidin-pretreated cells, the number of cells with nuclear condensation and fragmentation was markedly decreased (Figure 3C). These results suggest that hispidin protect palmitate-induced DNA condensation through alleviation of oxidative stress. 

### 2.4. Effect of Hispidin on Apoptosis-Associated Proteins

**Figure 4:**
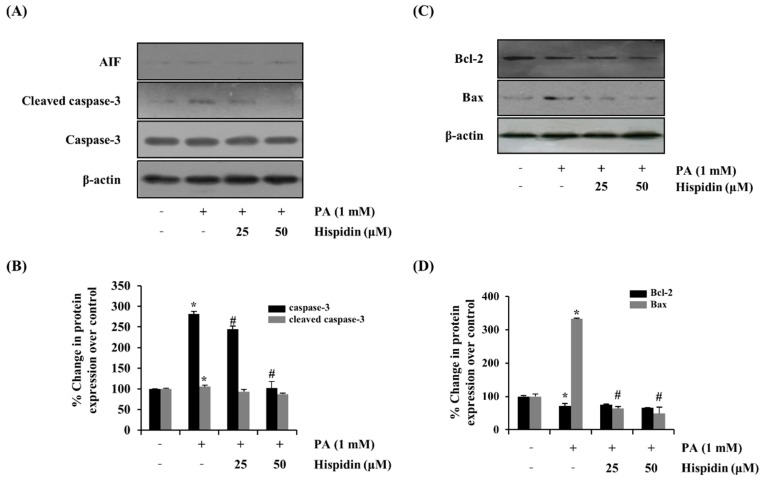
Effect of hispidin on the expression of apoptosis-associated proteins in palmitate-treated C2C12 myotubes. C2C12 myotubes were cultured with indicated concentration of hispidin for 8 h, and then exposed to 1 mM palmitate for 16 h. (**A**) The expression levels of AIF, cleaved caspase-3 and caspase-3 were assessed using western blot analysis. (**B**) Quantitative analysis of the band density of cleaved caspase-3 and caspase-3. (**C**) The expression levels of Bax and Bcl-2 were assessed using western blot analysis. (**D**) Quantitative analysis of the band density of Bax and Bcl-2. The equal loading of the total proteins in each sample was confirmed by the expression of *β*-actin. Data represent the mean ± standard error of three independent experiments. * *p* < 0.05, as compared with only BSA group, ^#^ *p* < 0.05, as compared with PA group. AIF, apoptosis-inducing factor; Bcl-2, B-cell lymphoma 2; Bax, Bcl-2-associated X protein.

**Figure 5:**
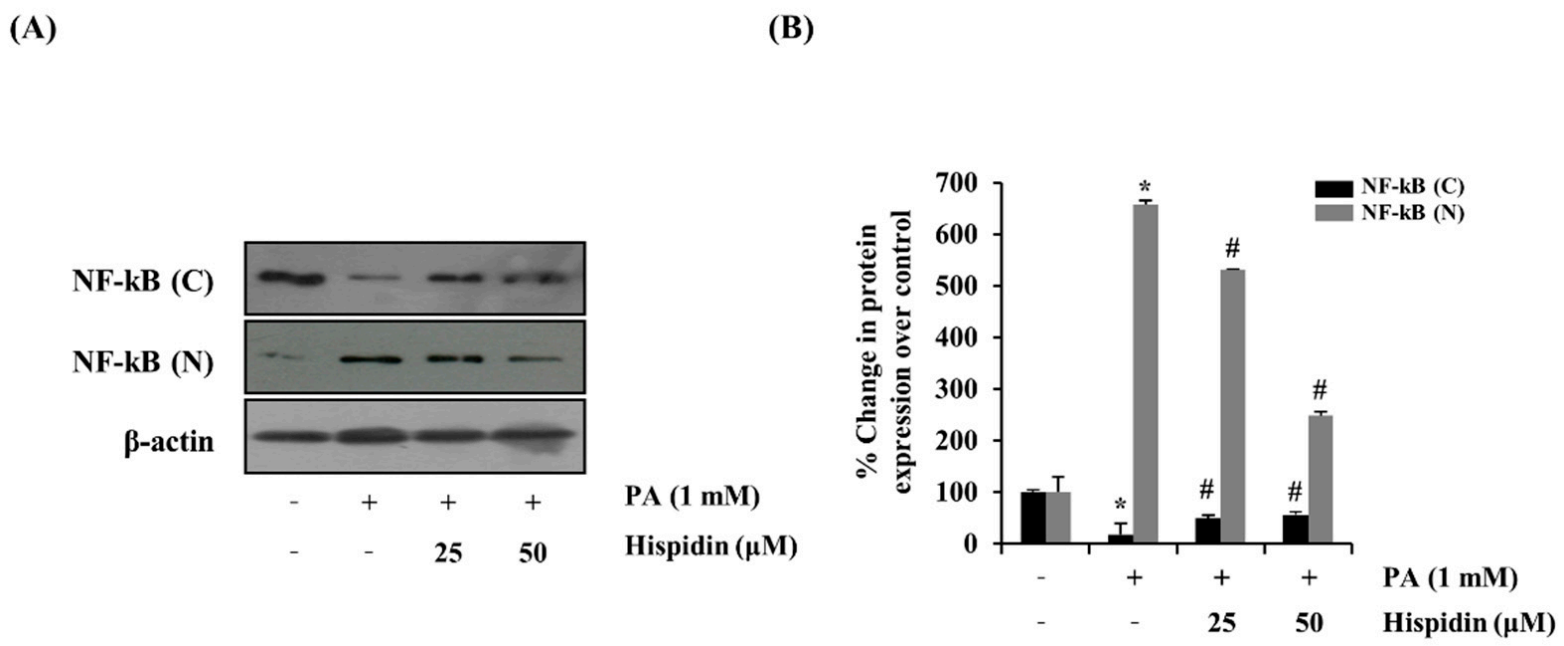
Effect of hispidin on the translocation of NF-κB in palmitate-treated C2C12 myotubes. C2C12 myotubes were cultured with indicated concentration of hispidin for 8 h, and then exposed to 1 mM palmitate for 16 h. (**A**) The expression levels of fractional NF-κB were assessed using western blot analysis. (**B**) Quantitative analysis of the band density of fractional NF-κB. The equal loading of the total proteins in each sample was confirmed by the expression of *β*-actin. Data represent the mean ± standard error of three independent experiments. * *p* < 0.05, as compared with only BSA group, ^#^ *p* < 0.05, as compared with PA group. NF-κB (N), nuclear fraction of nuclear factor-κB; NF-κB (C), cytosolic fraction of nuclear factor-κB.

To further determine the activation of caspase-3 by the treatment of hispidin, the activity of caspase-3 was analyzed by western blot. Hispidin (25 and 50 μM) dose-dependently reduced the palmitate-induced expression of caspase-3 compared with that of the palmitate-treated group. However, hispidin did not cause any change in AIF (apoptosis-inducing factor, Figure 4A). To further confirm that the mitochondrial apoptotic events were involved in palmitate-induced cell death, changes in the levels of Bax and Bcl-2 were analyzed by western blot. Hispidin decreased palmitate-induced expression of the pro-apoptotic protein Bax, but did not affect expression of the anti-apoptotic protein Bcl-2 (Figure 4B). Nuclear factor-kappa B (NF-κB) has been known to be involved in oxidative stress-induced cell death in different cell types; thus, the translocation of NF-κB from the cytosol to the nuclei was examined. As shown Figure 5, palmitate-induced nuclear translocation of NF-κB p65 was inhibited by pretreatment with hispidin (25 and 50 μM).

### 2.5. Discussion

In obese individuals, oversupply of lipids raises the circulating level of FFA and contributes to the development of type 2 diabetes [27]. High plasma FFA levels induce insulin resistance and tissue injury, such as pancreas and skeletal muscle [28,29]. It is generally proposed that mitochondrial dysfunction and subsequent apoptotic cell death are mediated by palmitate-induced ROS. Also, the accumulation of excess cellular FFA induce normal cell dysfunction; for example, FFA block beta-cell function by induction of glucose-dependent insulin secretory failure, increasing FFA reduces insulin-mediated suppression of hepatic glucose production, and cardiac steatosis leads to cardiomyopathy and impaired contractile function [9,28].

Oxidative stress-induced mitochondrial dysfunction and DNA condensation, which causes regulation of Bcl-family proteins, release of cytochrome c from mitochondria, and in turn, cleavage of caspase-3 [30]. As a result, cleavage of caspase-3 results in the execution of apoptosis. Because palmitate is one of the long chain fatty acids, high palmitate levels cause oxidative stress in C2C12 myotubes. In agreement with these reports, we observed that palmitate caused a dose- and time-dependent decrease in cell viability, intracellular ROS accumulation, and DNA condensation. Further, palmitate enhanced the expression of cleaved caspase-3, the release of cytochrome c, and the expression of the expression of pro-apoptotic Bax. However, this phenomenon was alleviated through the treatment of hispidin.

NF-κB plays a critical role in implementing pathogenesis in a number of inflammatory diseases including type 2 diabetes [31]. NF-κB regulates the expression of proinflammatory and anti-apoptotic genes. Some reports demonstrated that palmitate provoked an increase in cell death and insulin resistance via NF-κB translocation. Our results showed that palmitate increased NF-κB translocation. However, treatment with hispidin inhibited the translocation of NF-κB.

## 3. Experimental Section 

### 3.1. Materials

Dulbecco’s modified Eagle medium (DMEM), fetal bovine serum (FBS), penicillin/streptomycin (PS), and trypsin-EDTA were purchased from GIBCO (Grand Island, NY, USA). Dichlorodihydrofluorescein-diacetate (H_2_DCF-DA) was obtained from Molecular Probes (Carlsbad, CA, USA). Hispidin, albumin from bovine serum (fatty acid free), palmitate, Hoechst 33342, and mitochondria isolation kit were purchased from Sigma Biochemical (St. Louis, MO, USA). Caspase-3, cleaved caspase-3, NF-κB, and horseradish peroxidase (HRP)-linked anti-rabbit IgG were purchased from Cell Signaling (Beverly, MA, USA). Antibodies to beta-actin, Bax, Bcl-2, and HRP-linked goat anti-mouse IgG were purchased from Santa Cruz Biotechnology (Santa Cruz, CA, USA). All other chemicals were analytical grade.

### 3.2. Culture Condition and Differentiation

The C2C12 myoblasts were derived from a mouse skeletal myoblast cell line. The cells were obtained from American Type Culture Collection (ATCC, CRL-1772, Manassas, VA, USA). The C2C12 myoblasts were grown in DMEM (GIBCO, Grand Island, NY, USA) supplemented with 10% inactivated FBS and 1% penicillin/streptomycin and maintained at 37 °C in a humidified 5% CO_2_ incubator. The cells were cultured to ~60% confluence and harvested with 0.25% trypsin-EDTA. Cells were harvested and sub-cultured for an additional 48 h in DMEM. The C2C12 myotubes were obtained by culturing myoblasts in DMEM containing 10% heat-inactivated horse serum. Myotubes formation was achieved after 4 days of incubation, and the cells were used for subsequent experiments.

### 3.3. Preparation of Palmitate-BSA Complex and Treatment

The palmitate-BSA complex was prepared as described previously [32]. In brief, palmitate was dissolved in 100 mM NaOH by heating at 70 °C. After filtration, the solution was then diluted with 10% fatty acid free-BSA and stored at −20 °C. Palmitate treatments were performed with the concentrations indicated in the figure legends generally for 16 h. In all experiments, 2 h before the treatment with fatty acids, the culture medium was changed to serum-free DMEM.

### 3.4. Cytotoxicity of Hispidin and Palmitate

To confirm the cytotoxic effects of hispidin in C2C12 myotubes, cell viability was evaluated using the (3-[4,5-dimethylthiazol-2-yl]-2,5-diphenyltetrazolium) bromide (MTT) assay, which relies on the ability of viable cells to metabolically reduce the MTT tetrazolium salt to a purple formazan product, which can be quantified colorimetrically. Briefly, cells were pre-incubated in 12-well plates for 48 h at 37 °C in a humidified atmosphere of 5% CO_2_. After differentiation, the cells were incubated with hispidin (from 1 to 1000 μM) for 24 h. At the end of each exposure time, 50 μL of the MTT stock solution (5 mg/mL) in serum-free medium was added to each well and incubated for 2 h at 37 °C. After incubation, the cells were washed with PBS and the supernatants were aspirated. The formazan crystal in each well was dissolved in isopropyl alcohol, and the absorbance was determined at 570 nm using an ELISA microplate reader (model 550, Bio-Rad, Hercules, CA, USA). To confirm the cytotoxicity of palmitate in C2C12 myotubes, cell viability was evaluated using the MTT assay. Briefly, after differentiation, cells were incubated with various concentrations of palmitate for 16 h. At the end of each exposure time, 50 μL of the MTT stock solution (5 mg/mL) in serum-free medium were added to each well and incubated for 2 h at 37 °C. After incubation, the cells were washed with PBS, and the supernatants were aspirated. The formazan crystal in each well was dissolved in isopropyl alcohol, and the absorbance determined at 570 nm.

### 3.5. Detection of Myotube Formation

After treatment, to investigate palmitate-induced myotube atrophy and loss, the cells were fixed for 10 min with 4% formaldehyde, then stained with 1% crystal violet solution for 15 min, and washed three times with water. Images were taken under a microscope.

### 3.6. Intracellular ROS Scavenging Activity

To determine the effect of hispidin on palmitate-induced oxidative stress, the differentiated C2C12 myotubes were treated with various concentrations of hispidin for 8 h, followed by the addition of 1 mM palmitate to each well for 16 h. After treatment, cells were incubated with 5 μM H_2_DCF-DA solution in phosphate buffered saline (PBS, pH 7.38), and fluorescence was measured at excitation and emission wavelengths of 485 nm and 535 nm, respectively, using a microplate fluorometer. For image analysis of the production of intracellular ROS, after treatment, H_2_DCF-DA solution was added to each well of the plate, which was incubated for 2 h at 37 °C. Images of the stained cells were collected using a fluorescence microscope (Nikon, Tokyo, Japan). 

### 3.7. Detection of Nuclear Morphology

The nuclear morphology of the cells was evaluated using the cell-permeable, DNA-specific fluorescent dye, Hoechst 33342. Cells with homogeneously stained nuclei were considered viable, whereas the presence of chromatin condensation and/or fragmentation was indicative of apoptosis. The C2C12 myotubes were seeded in coverslip-loaded 6-well plates. Then, 24 h after plating, the cells were treated with various concentrations of hispidin for 8 h and then 1 mM palmitate was added for 16 h. Then, 5 μg of Hoechst 33342 (stock 10 mg/mL) was added to each well, followed by 10 min of incubation at room temperature. Images of the stained cells were collected using a fluorescence microscope (Nikon), in order to examine the degree of nuclear condensation.

### 3.8. Preparation of Fractional Proteins

Nuclear extracts were prepared by lysing nuclei in a high salt buffer supplemented with protease and phosphatase inhibitors using a nuclear extraction kit (Panomics Inc., Fremont, CA, USA) according to the manufacturer’s protocol. Protein concentrations were quantified by the Bio-Rad protein assay (Bio-Rad Laboratories).

### 3.9. Western Blot Analysis

After various treatments, cells were washed in 1× PBS and lysed in lysis buffer (10 mM Tris-HCl [pH 7.5], 10 mM NaH_2_PO_4_/NaHPO_4_ [pH 7.5], 130 mM NaCl, 1% TritonX-100, 10 mM NaPPi, 1 mM phenyl methyl sulphonyl fluoride, 2 μg/mL pepstatin A) for 30 min on ice. Lysates were centrifuged at 12,000 *g* for 20 min at 4 °C. The supernatant was collected, and the protein content of the supernatant was measured using a Bio-Rad protein assay kit before analysis. The fractional protein samples were loaded at equal amounts of protein/lane, separated by SDS-PAGE on a 10%–15% gel, and transferred to nitrocellulose membranes (NC membrane, 0.2 μm; Bio-Rad). Membranes were blocked with 5% nonfat powdered milk in 1× Tris-buffered saline containing 0.1% Tween 20 (TBS-T) for 1 h and then incubated with primary antibodies at 4 °C overnight. Finally, the membranes were treated with horseradish peroxidase-linked secondary antibodies for 1 h at 4 °C. The membranes were washed with TBS-T after each antibody-binding reaction. Detection of each protein was performed using an enhanced chemiluminescence kit (Millipore Co., Billerica, MA, USA). Quantitation analyses for Western blotting were performed using the Image J densitometry software program.

### 3.10. Statistical Analysis

Data were expressed as means ± standard error (SEM), and the results were taken from at least three independent experiments performed in triplicate. The data were analyzed by Student’s *t*-test to evaluate significant differences. A *p* value < 0.05 was considered statistically significant.

## 4. Conclusions 

In conclusion, our study indicated that hispidin effectively prevented palmitate-induced lipotoxicity in myotubes. Specifically, hispidin improve intracellular ROS concentration and DNA condensation. Moreover, hispidin inhibit apoptosis-associated protein in C2C12 myotubes. Hence, we provided evidence that hispidin may have the therapeutic potential as an anti-diabetic drug. 
